# FSH stimulates expression of the embryonic gene HMGA2 by downregulating let-7 in normal fimbrial epithelial cells of ovarian high-grade serous carcinomas

**DOI:** 10.3892/etm.2012.794

**Published:** 2012-02-11

**Authors:** XU-YIN ZHANG, JING-XIN DING, XIANG TAO, KE-QIN HUA

**Affiliations:** Department of Obstetrics and Gynecology, Obstetrics and Gynecology Hospital, Fudan University, Shanghai 200090, P.R. China

**Keywords:** HMGA2, let-7, high-grade serous carcinoma, FSH

## Abstract

FSH may increase the risk of ovarian malignancy and play a key role in ovarian carcinogenesis, although the mechanism(s) are undefined. HMGA2 overexpression has been observed to be an early genetic event in tumorigenesis. The present study was designed to investigate the effect of FSH on let-7, HMGA2 and p53 expression in normal fimbrial epithelial cells of ovarian high-grade serous carcinomas (HGSCs). A primary human Fallopian tube (FT) fimbrial epithelium *ex vivo* culture system of low-grade serous carcinomas (LGSCs) and HGSCs was established. The levels of HMGA2, let-7, p53 and FSHR were evaluated by western blotting and reverse transcription (RT)-PCR. Treatment with FSH significantly increased HMGA2 expression in a time-dependent manner and the let-7 expression levels decreased gradually over time in the normal fimbrial epithelial cells of HGSCs. However, we did not observe similar results in LGSCs. In addition, knockdown of let-7 suppressed HMGA2 expression. p53 was not detected in the normal fimbrial epithelial cells before or after FSH administration. Our results indicate that FSH increases the expression of HMGA2 by downregulating the expression of let-7 in normal fimbrial epithelial cells of HGSCs, but no occurrence of p53 mutation. The susceptibility of fimbria to FSH in HGSCs compared with those in LGSCs is different.

## Introduction

Epithelial ovarian cancer (OC) is the most common cause of mortality from gynecological malignancy and the majority of ovarian carcinomas are of serous type. Serous carcinomas are further subclassified as high- or low-grade, based on histological features (1). The group designated ‘atypical proliferative serous tumor (APST)’ performed in a benign fashion, and a second, smaller group designated ‘micropapillary serous carcinoma (MPSC)’ or ‘noninvasive low-grade serous carcinoma’ performed as a low-grade malignant tumor (2). The latter subset was closely associated with invasive low-grade serous carcinoma (LGSC) and the investigators proposed that MPSC was the immediate precursor of LGSC. LGSC is a distinct entity that differs from high-grade serous carcinoma (HGSC) in several ways. For example, LGSC has specific mutations in genes such as BRAF and KRAS.

Compelling evidence is emerging that numerous high-grade ovarian serous carcinomas originate from the epithelium of the distal fimbrial portion of the Fallopian tube (FT) (3). The FT mucosa was suggested as a strong candidate for the primary source of pelvic (ovarian, tubal or peritoneal) serous carcinoma. Serous tubal intraepithelial carcinoma (STIC) has been implicated in the origins of not only HGSC but also serous carcinomas and primary peritoneal carcinomas. It has been proposed that the earliest neoplastic change begins in secretory-type cells (3). Further evidence supporting the proposal that STICs are precursors was the identification of STICs in females without OC as well as the presence of identical p53 mutations in STICs and concomitant ovarian HGSCs, indicating a clonal relationship between them (4).

HMGA2, a high-mobility-group AT-hook (HMGA) protein, is a non-histone DNA-binding factor that binds to AT-rich sequences in the minor groove of the DNA helix. HMGA2 is expressed in embryonic tissue, but not in the majority of adult tissues and is an important regulator for cell growth, differentiation, apoptosis and malignant transformation (5). HMGA2 overexpression has been observed to be an early genetic event in tumorigenesis. The expression of HMGA2 is regulated by microRNA. Previous studies show that let-7s specifically repress HMGA2 expression both *in vivo* and *in vitro*, revealing the regulatory role of let-7 in HMGA2 expression (5–7). Downregulation of let-7s is common in OC. OC patients with high HMGA2 and low let-7 expression in cancerous cells had a lower survival than patients with a low HMGA2/high let-7 ratio (6,7).

Levels of ovarian and peritoneal FSH and LH appear to be elevated in OC patients (8). Although the mutagenic effect of FSH remains controversial, the proliferative effect has been demonstrated in ovarian surface epithelium (OSE), in OC *in vitro* and in several OC cell lines in a dose- and time-dependent manner *in vitro*(9,10). However, little is known about the exact mechanism of FSH stimulation in the tumorigenesis of OC. In addition, the correlation of HMGA2 expression, p53 mutation and FSH, is poorly understood.

The purpose of the present study was to investigate the effect and mechanism of FSH on let-7, HMGA2 and p53 expression in the normal fimbrial epithelial cell of HGSCs and reveal the different susceptibilities to FSH of fimbria in HGSCs and LGSCs.

## Materials and methods

### Tissue samples

Fresh FT fimbria specimens were obtained from the Obstetrics and Gynecology Hospital of Fudan University (Shanghai, China) with the approval of the review board of the Obstetrics and Gynecology Hospital, Fudan University. The fimbrial tissues used in this study were collected from surgical procedures for 18 cases of HGSCs and 16 cases of LGSCs between January 2011 and December 2011 in the Obstetrics and Gynecology Hospital of Fudan University. None of the patients had a history of other neoplasms or had undergone radiotherapy, chemotherapy, hormone replacement therapy, immunotherapy or any other therapy prior to surgery. Following surgery, 2 pathologists reviewed the hematoxylin and eosin-stained sections from each specimen to exclude STIC and fimbria involvement for each case. p53 immunohistochemistry was performed to exclude positive cases. The histological diagnosis and tumor grade of HGSCs and LGSCs were based on the conventional criteria (1). In addition, clinical characteristics, including age and disease stage are listed in Table I.

The 15 cases of fimbrial tissues used for the control group in this study were collected from surgical procedures for benign gynecological indications. Cases of inflammatory disease, infection and extensive adhesions were excluded. The primary human FT epithelium *ex vivo* culture system was completed according to a previous study (11,12).

### Western blot analysis

Cell lysates from the culture were collected and quantified using the BCA method. Following 8, 12 and 15% denaturating sodium dodecyl sulfate-polyacrylamide gel electrophoresis, 30 μg of protein lysates was separated from the gel and transferred to a nitrocellulose filter. The membranes were sealed with PBS containing 5% non-fat milk for 1 h at room temperature and then sealed with a primary antibody (anti-HMGA2 antibody, 1:50, Santa Cruz Biotechnology, Inc., Santa Cruz, CA, USA; anti-FSHR antibody, 1:400, Lab Vision Co., Fremont, CA, USA; anti-p53 antibody, 1:1000, Abcam, Cambridge, MA, USA) overnight at 4°C. The following day, the membranes were mixed with HRP-conjugated secondary antibodies for 1 h at 37°C. GAPDH was used as a loading control. The signal was detected with an enhanced chemiluminescence assay (PerkinElmer, Waltham, MA, USA) and the protein was analyzed semiquantitatively using the software Quantity One (Bio-Rad, Hercules, CA, USA).

### RNA extraction and reverse transcription (RT)-PCR

The levels of let-7b microRNA were determined by RT-PCR. Total cellular RNA was extracted from cells using the TRIzol reagent (Invitrogen, Carlsbad, CA, USA) according to the manufacturer’s instructions. cDNA was synthesized from 2 μg RNA using a reverse transcription kit (Promega, Madison, WI, USA) and PCR primers by Yingjun Biotechnology Corporation (Shanghai, China). The mature let-7b (Applied Biosystems, Carlsbad, CA, USA) sequence was 5′-UGAGGUAGUAGGUUGUGUGGUU-3′. The conditions for amplification were as follows: one cycle at 94°C for 5 min, followed by 50 cycles at 94°C for 30 sec, 57°C for 30 sec and 70°C for 30 sec. In total, 20 μl PCR product was used for agarose electrophoresis.

### FSH stimulation

FSH was purchased from Sigma Chemical Co. (St. Louis, MO, USA). GAPDH monoclonal antibody was purchased from Kangchen Bioengineering Corporation (Shanghai, China). The Fallopian tube epithelium (FTE) cells were plated at 4×10^4^ or 4×10^5^ and 1×10^4^ or 1×10^5^ cells per well onto 96-well or 6-well plates, respectively. Twenty-four hours after plating, RPMI-1640 medium without serum was replaced and the cells were serum-starved for 18 h. The cells were then stimulated with FSH at 40 mIU/ml for different time periods (up to 120 min for signaling or up to 24 h for protein expression), PBS was used as a control. Transfected cells were also starved for 18 h and then stimulated with FSH at 40 mIU/ml for an additional 24 h. The cells were then harvested and the proteins were extracted for western blot analysis.

### Anti-let-7b transfection

FTE cells of HGSCs were transfected in 12-well plates with 60 pmol of anti-miR let-7b or equivalent amounts of negative control #1 miRNA inhibitor (Ambion, Austin, TX, USA) using Lipofectamine 2000 (Invitrogen, Carlsbad, CA, USA) according to the manufacturer’s instructions and cells were incubated for 48 h after transfection.

### Statistical analysis

The results of the experiments were analyzed using the χ^2^ test for positive rate comparison and one-way analysis of variance for the other comparisons. P<0.05 was considered to indicate a statistically significant result. The SPSS software program (version 12.0; SPSS, Inc., Chicago, IL, USA) was used for all statistical analysis.

## Results

### The expression of HMGA2, let-7, p53 and FSHR in FTEs

**Figure 1:**
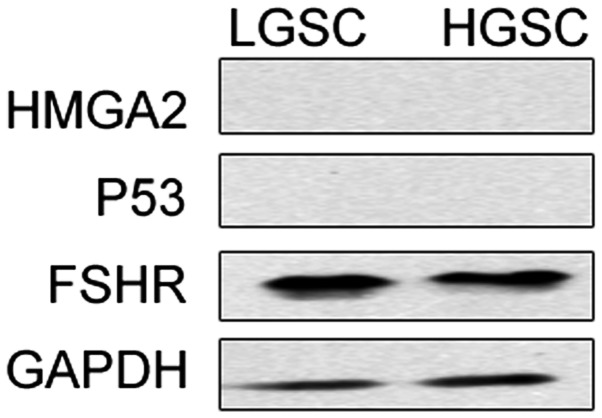
HMGA2 and p53 expression were not detected by western blot analysis in LGSCs and HGSCs. FSHR mRNA expression detected by western blot analysis was observed in 100% of the FTE cells of HGSCs and LGSCs. GAPDH was used as a loading control. HGSC, high-grade serous carcinoma; LGSC, low-grade serous carcinoma; FTE, Fallopian tube epithelium.

HMGA2, let-7, p53 and FSHR had similar expression levels in FTE cells of LGSCs and HGSCs. This result was confirmed by RT-PCR and western blot analysis. All 34 samples expressed let-7b. HMGA2 and p53 expression were not detected in any samples. FSHR mRNA expression revealed by western blot analysis was observed in 100% of the FTE cells of HGSCs and LGSCs (Fig. 1).

### FSH increases expression of HMGA2 and decreases expression of let-7 in normal fimbria of HGSCs

**Figure 2:**
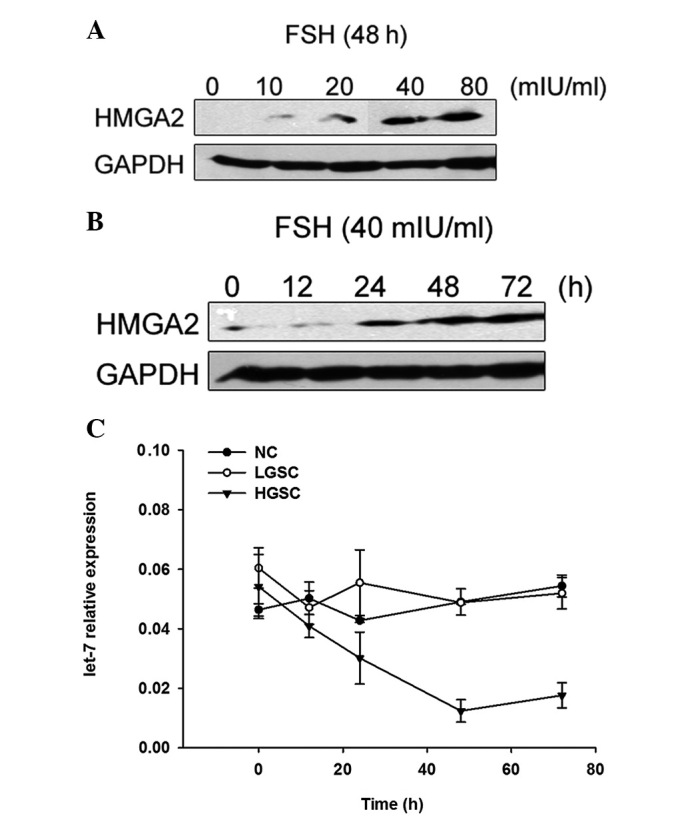
FSH stimulation of HMGA2 expression in FTE cells of HGSCs. The level of HMGA2 protein expression was determined using western blot analysis following FSH stimulation at different doses and time points. (A) Dose response: HMGA2 protein expression in FTE cells of HGSCs after stimulation with 0 to 80 mIU/ml FSH. (B) Time response: HMGA2 protein expression in FTE cells of HGSCs after stimulation with 40 mIU/ml FSH for 0–72 h. (C) Decreased let-7 expression in HGSCs after stimulation with FSH treatment. The level of let-7 expression was determined using RT-PCR following stimulation with 40 mIU/ml FSH at different time points and was lowest from 48 h after stimulation with 40 mIU/ml FSH. let-7b expression in FTE cells of the LGSCs by FSH stimulation was not markedly altered. NC, negative control; HGSC, high-grade serous carcinoma; LGSC, low-grade serous carcinoma; FTE, Fallopian tube epithelium.

We stimulated the FTE cells with FSH at various concentrations and for various time courses. Western blot analysis showed that HMGA2 expression levels increased gradually in addition to the FSH concentration in FTE cells of the HGSCs, peaked at an FSH concentration of 40 mIU/ml, and then began to decline slightly, indicating a dose-dependent correlation (Fig. 2). When FTE cells of the HGSCs were stimulated with 40 mIU/ml of FSH for 0, 12, 24, 48 or 72 h, HMGA2 expression in the 24, 48 and 72 h groups of cells increased in comparison to the control group, indicating a time-dependent correlation. HMGA2 expression peaked from 48 h after stimulation with 40 mIU/ml FSH (Fig. 2). Notably, we observed that the let-7 expression levels decreased gradually with time and an inverse correlation between the expression of let-7b and HMGA2 in FTE cells of the HGSCs was observed following FSH stimulation (r=−0.55, P=0.006). p53 was not detected in the normal fimbrial epithelial cells before or after FSH administration. However, we did not observe changes of HMGA2, let-7b or p53 in FTE cells of the LGSCs due to FSH stimulation. The results demonstrated that the susceptibility of fimbria of LGSCs and HGSCs was different.

### FSH stimulates HMGA2 expression by downregulating let-7

We observed that FSH stimulation of the FTE cells of HGSCs significantly increased the expression of HMGA2 and decreased the expression of let-7b, but had no effect on let-7b and HMGA-2 of LGSCs. To confirm that let-7 regulated HMGA2 expression, we pretreated the FTE cells of HGSCs with anti-miR let-7b transfection for 48 h. HMGA2 expression was detected and p53 was absent, as demonstrated by western blot analysis. These data indicate that let-7 regulated HMGA2 expression and FSH stimulates HMGA2 expression by down-regulating let-7.

## Discussion

The observations that FSH increases the risk of ovarian malignancy and that pregnancies or oral contraceptives protect the ovaries by suppressing FSH secretion led to numerous studies (13). The majority of studies addressing the role of FSH observed that it has a growth stimulation effect in normal or immortalized ovarian surface epithelial cells (10). SV-40 transformed benign ovarian epithelial tumor cells and certain OC cells in a dose- and time-dependent manner *in vitro*, as observed in our previous study that examined the role of FSH in ovarian carcinogenesis (14).

Since a comparable amount of FSHR is also present in the FT, including the fimbriated end (15), certain high grade ovarian epithelial cancers with likely tubal origin do not negate the role of FSH in ovarian carcinogenesis. Our results showed that FSHR mRNA expression revealed by western blot analysis was observed in 100% of the FTE cells of HGSCs and LGSCs and was consistent with the literature. The FTE cells show a limited ability to resolve the damage over time, potentially leaving the cells more susceptible to the accumulation of additional mutagenic injury (11). However, susceptibility to hormones, such as FSH, is not clear.

In this study, we observed that FSH stimulation of the FTE cells of HGSCs significantly increased the expression of HMGA2 and decreased the expression of let-7b, but had no effect on that of LGSCs. This suggested significant biological differences in the behavior of the fimbria in high-grade and low-grade ovarian serous cancers (OSCs) and we presumed that the susceptibility to FSH of fimbria of high- and low-grade OSCs are different. In addition, we detected FSHR expression in all FTE cells of HGSCs and LGSCs. It is likely that FSH regulated let-7b via FSHR. However, thus far little is known about the mechanism of FSH stimulation of let-7 and HMGA2 expression and it was lack of investigation *in vitro* and *in vivo* to confirm the mechanism.

HMGA2 overexpression has been associated with tumor growth, differentiation, metastasis, unfavorable outcome and resistance to treatment (5). Silencing of HMGA2 expression in OC cells has been reported to have a therapeutic effect on OC growth (6). HMGA2 upregulation occurs early during OC progression before the tumors begin to metastasize both in human patients and in an OC mouse model (7).

Serous intraepithelial carcinoma in the FT has a high rate and level of HMGA2 overexpression in addition to p53-dominant mutations (16). The immunohistochemistry results indicate that HMGA2 overexpression is an early event in the tumorigenesis of high-grade papillary serous carcinoma. In addition, it suggests that the later event of HMGA2 over-expression is after p53 mutations. However, we observed the expression of HMGA2 but no occurrence of p53 mutation with the stimulation of FSH. The mutation of p53 may have caused the expression change of HMGA2. The detailed mechanism requires further research. Levanon *et al*(17) proposed a sequential model of HGSC, progressing from precursor with p53 signature, to STIC and then to invasive carcinoma (3,17). However. the cause of the p53 signature remains unknown.

We identified HMGA2 as a significant molecule for future studies of HGSCs due to its potential use in the early detection of disease and its functional role in tumorigenesis. FSH likely plays a key role in the early carcinogenesis and has no effect in the advanced process.

Members of the let-7/miR-98 family are induced late in mammalian embryonic development to suppress the expression of embryonic genes that are not expressed in the adult organism. Reported let-7 targets include RAS, c-myc and HMGA2. let-7 is frequently downregulated in human neoplasms, suggesting that embryonic target genes of let-7 are upregulated in cancer.

let-7 expression causes degradation of HMGA2 mRNA. The efficient degradation of HMGA2 mRNA may be due to the high degree of complementarity of let-7 to certain let-7 seed matches present in the HMGA2 untranslated region (3′-UTR) (18–23). There was significant downregulation of let-7b in high-grade papillary serous carcinomas in comparison to matched FTE. Therefore loss of let-7 expression plays a key role in the regulation of FTE cells (24).

In the present study, we observed that the let-7 expression levels decreased gradually over time and an inverse correlation between the expression of let-7b and HMGA2 in FTE cells of the HGSCs stimulated by FSH was present. A previous study showed that HMGA2 was a direct target for let-7 in human cancer cell lines and let-7 regulated HMGA2 expression in OC and predicts disease progression (20).

In conclusion, our results suggest that FSH stimulation of HMGA2 expression is mediated by let-7. Further studies to understand the role of FSH in tumorigenesis of FTE cells at cellular and molecular levels are required, as this may elucidate the etiology of OC development.

## Figures and Tables

**Figure 3:**
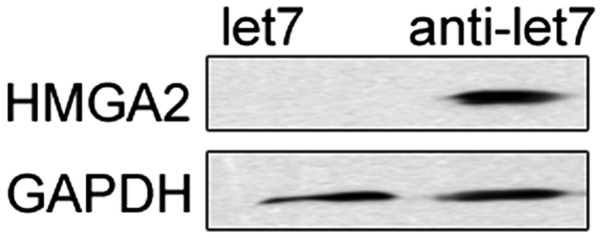
Transfected with anti-miR let-7b, HMGA2 expression was detected by western blot analysis in HGSCs. GAPDH was used as a loading control. HGSC, high-grade serous carcinoma.

**Table I:** Clinical characteristics of the patients.

Characteristic	HGSC (n)	LGSC (n)
Total	18	16
Patient age (years)		
≤50	6	7
>50	12	9
CA125 (U/ml)		
≤500	3	6
>500	15	10
Disease stage		
I–II	3	5
III–IV	15	11
Residual disease		
<1 cm	14	15
≥1 cm	4	1

HGSC, high-grade serous carcinoma; LGSC, low-grade serous carcinoma.
